# Genome-wide identification, characterization and *in-silico* expression of AINTEGUMENTA-LIKE family in *Eucalyptus grandis*

**DOI:** 10.3389/fpls.2026.1798071

**Published:** 2026-05-01

**Authors:** Jiahao Zeng, Shiying Zhang, Yongxiong Fan, Xiaochi Yu, Jiye Tang, Ai-Min Wu, Xianhai Zhao

**Affiliations:** 1 Guangdong Key Laboratory for Innovative Development and Utilization of Forest Plant Germplasm, College of Forestry and Landscape Architectures, South China Agricultural University, Guangzhou, China; 2 State Key Laboratory of Tree Genetics and Breeding, Key Laboratory of State Forestry Administration on Tropical Forestry, Research Institute of Tropical Forestry, Chinese Academy of Forestry, Guangzhou, China

**Keywords:** AIL family, *Eucalyptus grandis*, functional analysis, genome-wide identification, somatic embryogenesis

## Abstract

The AINTEGUMENTA-LIKE (AIL) family is a current research hotspot in plant molecular biology, primarily focusing on its potential applications in crop genetic improvement. It plays roles in plant embryogenesis, meristem maintenance, organ growth regulation, and cross-talk with hormone signaling pathways. However, the members and functions of *AIL* genes in *Eucalyptus grandis* remain uncharacterized. In this study, a genome-wide characterization of the AIL family in *E. grandis* was performed using bioinformatics methods, resulting in the identification of 19 AIL members (*EgAIL1A*–*EgAIL16*). All members contain the characteristic APETALA 2(AP2) conserved domain and were classified into the AP2/ERF superfamily. Phylogenetic analysis revealed that these genes are most closely related to *PtrAIL*s from *Populus trichocarpa*, and they clustered into five subgroups. Based on *cis*-acting element analysis, tissue-specific expression profiles, and stress treatment assays, EgAIL3 was identified as a positive regulator of salt tolerance; while EgAIL16 is predicted to be involved in regulating early root and leaf development regulation and contains an endodermis-specific negative regulatory element. This study provides a foundation for understanding the AIL family and a theoretical basis for refining *E. grandis* somatic embryogenesis.

## 1 Introduction

*Eucalyptus grandis*, native to Australia, is considered one of the most extensively cultivated fast-growing hardwood species worldwide (Trueman et al., 2018). Its wood and biomass hold significant commercial value for pulp production, papermaking, and bioenergy (Silva et al., 2003). As a representative fast-growing tree, with an annual production exceeding 200 million tons, it plays a crucial role in global plantation forestry (Long, 2024). However, technical bottlenecks in the efficient propagation of superior, mature genotypes persist. Current somatic embryogenesis (SE) protocols for *E. grandis* involve complex *in vitro* stress treatments, such as high hormone concentrations and osmotic stress. These methods face challenges including low induction efficiency of embryogenic callus (Pinto et al., 2008), poor synchronization of somatic embryo development (Pinto et al., 2002), and a high frequency of abnormal plantlet development (Wang et al., 2024), which severely limit the large-scale clonal propagation of elite germplasm (Abiri et al., 2020). SE, an important *in vitro* regeneration pathway utilizing cell totipotency, plays a key role *in vitro* propagation (Xiong et al., 2022) and is a pivotal technology for overcoming this bottleneck. Somatic embryos generated via SE exhibit bipolar development and can directly regenerate into intact plantlets without a distinct rooting phase (Von Arnold et al., 2002). Due to its high-efficiency-regeneration characteristics, SE technology can provide technical support for the large-scale propagation of superior *E. grandis* genotypes, long-term germplasm conservation, and molecular breeding. Furthermore, it can facilitate the generation of haploids and doubled haploids through somaclonal variation and *in vitro* mutagenesis (Long, 2024) and promote the development of hybrid combinations with superior genotypes, laying the foundation for rapid breeding of high-yield, high-quality *E. grandis* (Sánchez Romero, 2021). Notably, studies have confirmed that transcription factors such as BABY BOOM (BBM), WUSCHEL (WUS), and PLETHORA 1 (PLT1) are key regulators of SE (Haecker et al., 2004; Horstman et al., 2017). Most of these transcription factors belong to the AINTEGUMENTA-LIKE (AIL) family, where they perform core regulatory functions. Therefore, investigating the AIL family in *E. grandis* provides, to some extent, a theoretical foundation for breaking through this propagation bottleneck.

As core regulators of SE, the AIL family belongs to the AP2 subfamily within the phylogenetically conserved AP2/ERF superfamily (Kim et al., 2006). The AP2/ERF superfamily is plant-specific and characterized by the presence of a conserved AP2 domain consisting of 60–70 amino acid residues. The secondary structure of this domain includes a β-sheet region and antiparallel α-helices. The AP2 subfamily can be further divided into three evolutionary lineages based on nuclear localization signals, amino acid motifs, and other conserved motifs: euAP2, basal ANT, and euANT (Shigyo et al., 2006; Hong et al., 2020). Members of the AIL subfamily are crucial for maintaining meristematic activity and regulating the stem cell niche in plants (Horstman et al., 2015; Kareem et al., 2015). This subfamily includes ANT, AIL1, PLT1, PLT2, AIL6/PLT3, PLT7, BBM, and AIL5/PLT5 (Nogueira et al., 2020). AIL genes play a central role in regulating somatic embryo formation, clonal propagation, and haploid induction. They promote cell proliferation and regeneration, induced adventitious root formation, and enhanced genetic transformation efficiency (Wang et al., 2020; Liu et al., 2023; Skinner et al., 2023; Shi and Ma, 2025). Specifically, *PLT1* and *PLT2* encode AP2-type transcription factors and act as core regulators of the embryonic stem cell niche during embryo development, being essential for SE induction in *Arabidopsis* (Tsuwamoto et al., 2010). These genes are expressed in the basal region of the embryo and are involved in the formation of the hypocotyl, root primordium, and root stem cells. Ectopic expression of *PLT1* and *PLT2* in the shoot apical meristem can also induce the formation of these structures (Aida et al., 2004). In other plant species, extensive research has confirmed the regulatory roles of AIL family genes. For example, *PLT* genes in *Arabidopsis* were crucial for the establishment and maintenance of root stem cells (Shimotohno et al., 2018), while *PLT1*, *PLT2*, and *AIL5/PLT5* influenced the formation of embryonic body plan (Aida et al., 2004; Tsuwamoto et al., 2010). In apple (*Malus domestica*), overexpression of *MdAIL5* modulates embryogenic callus formation and significantly improves the efficiency of adventitious root and shoot regeneration (Liu et al., 2023). Furthermore, the *BBM* gene also serves as a key marker and transcriptional regulator during embryo development (Khanday et al., 2023). It plays a central role in multiple processes, including inducing SE, parthenogenesis, haploid induction, promoting cell proliferation and regeneration, and enhancing genetic transformation efficiency (Skinner et al., 2023; Shi and Ma, 2025). Heterologous expression of *BBM* in *Brassica napus* and *Arabidopsis* can directly induce somatic embryo formation without requiring exogenous hormones (Li et al., 2021). In *Paeonia ostii*, *PoBBM* formed a synergistic regulatory network with *PoAHL15* to positively regulate early SE (Zhu, 2025). These findings suggest that members of the *E. grandis* AIL family may also play critical roles in embryogenic callus induction, synchronization of somatic embryo development, and the initiation of SE under non-stress conditions. These findings provide potential molecular targets for addressing the key bottlenecks in *E. grandis* SE technology. Consequently, *AIL* overexpression is widely regarded as an important tool and a valuable strategy in plant biotechnology. Investigating its mechanisms for inducing embryonic development and regulation of adventitious shoot regeneration holds significant potential for application in forestry and woody plant regeneration (Lutz et al., 2015; Han et al., 2022).

While certain members of the AIL gene family have been established as key regulators of SE in other species, capable of integrating hormonal signals to control embryonic development and organ regeneration, significant gaps remain in the study of the AIL family in *E. grandis* compared to other investigated species. Currently, the functional annotation and characterization of AIL family members in *E. grandis* are incomplete. Their expression patterns, functional differentiation, and the mechanisms underlying the regulation of SE and clonal propagation remain unclear and insufficiently studied. Current *E. grandis* SE protocols predominantly rely on complex *in vitro* stress treatments. However, recent studies have shown that ectopic expression of the *BBM-BAR1* gene can induce microspore embryogenesis under stress-free conditions (Shi et al., 2025). Furthermore, the *PLT3b* gene from *Aquilaria sinensis* significantly promoted callus growth, and the *PLT2* gene exhibited the most pronounced effect on promoting root regeneration in *Arabidopsis* (Wang, 2024). These findings provide novel targets for improving *E. grandis* seed germination rates and facilitating the rapid clonal propagation of elite woody plant germplasm, thereby highlighting the necessity of investigating the AIL family in *E. grandis*.

Therefore, this study was conducted to investigate the molecular regulatory basis of highly efficient SE and to identify candidate breeding genes in *E. grandis* through a genome-wide identification and bioinformatic analysis of its AIL family. It aimed to characterize the evolutionary relationships, and conserved domains of EgAIL proteins. Furthermore, we analyzed the expression profiles of *EgAIL* genes across various tissues and key SE stages to screen for key members involved in regulating *E. grandis* SE. This research will address the current knowledge gap regarding the AIL family in *E. grandis*, providing a theoretical foundation and genetic resources for understanding the molecular mechanisms of its clonal propagation and for enhancing breeding efficiency through genetic engineering.

## 2 Materials and methods

### 2.1 Data collection

The reference genome sequence and its corresponding annotation for *E. grandis* were obtained from the Phytozome database (Myburg et al., 2014). Corresponding *AIL* gene sequences from *Arabidopsis thaliana* (*At4g37750*, *At1g72570*, *At5g17430*, *At3g20840*, *At1g51190*, *At5g57390*, *At5g10510*, and *At5g65510*) were sourced from The Arabidopsis Information Resource (TAIR) (Lamesch et al., 2012; Berardini et al., 2015) (https://www.arabidopsis.org/; accessed on 13 July 2025). Genomic data for the bryophyte *Physcomitrella patens* were obtained from the NCBI Genome portal (Tuskan et al., 2006; Banks et al., 2011) (https://www.ncbi.nlm.nih.gov/genome/?term=; accessed on 13 July 2025). In addition, for comparative analysis, genome assemblies for the representative species (*Selaginella moellendorffii*, *Chlamydomonas reinhardtii*, and *P. trichocarpa*) were retrieved from EnsemblPlants (https://plants.ensembl.org/index.html; accessed on 13 July 2025). Collectively, these genomic datasets laid the foundation for subsequent gene family analyses.

### 2.2 Comparative genomics and functional annotation of the AIL family in *E. grandis*

The predicted protein sequences of the identified *EgAIL* genes were analyzed using TBtools software (Chen et al., 2022). Comparative analysis of AIL protein sequences between *A. thaliana* and *E. grandis* was conducted using the “Blast Compare Two Seqs” module in TBtools. Default Blastp parameters were applied, specifically with an E-value cutoff of 1e^-5^, a maximum of 500 hits, and 250 alignments. These IDs were subsequently imported into the Fasta Extract tool of TBtools to retrieve the corresponding protein sequences, generating an initial set of candidates of EgAIL family. All candidates were further screened using the NCBI Conserved Domain Database (CDD) (accessed on 14 July 2025) to retain only those sequences that contained two AP2 domains. Ultimately, 19 EgAIL family members were identified. Multiple biophysical properties of the 19 identified AIL proteins were calculated using the “Protein Parameter Calc” tool in TBtools. The analyzed parameters included amino acid length, molecular weight, isoelectric point (pI), instability index, and grand average of hydropathicity (GRAVY).

### 2.3 Evolutionary relationships and phylogenetic diversification of *AIL* genes in *E. grandis*

For the phylogenetic reconstruction of the AIL family, protein sequences from five key representative species (specifically, *A. thaliana*, *S. moellendorffii*, *P. patens*, *C. reinhardtii*, and *P. trichocarpa*) were compiled using the same procedure in screening *EgAILs*. These sequences, along with the identified EgAILs, were subjected to multiple sequence alignment and homology evaluation. Initially, the homologous protein sequences corresponding to these species were retrieved through TBtools. A multiple sequence alignment was subsequently generated using the MUSCLE algorithm integrated in MEGA 11.0 (Tamura et al., 2021) with default alignment parameters. Phylogenetic reconstruction was performed using the Neighbor-Joining method, employing 1000 bootstrap replicates and all other parameters set to default values. To facilitate topological examination and enhance graphical visualization, the resulting phylogenetic tree was uploaded to the Interactive Tree of Life (iTOL) platform for further annotation and customization (Letunic and Bork, 2024).

### 2.4 Gene structure and conserved motifs analysis of EgAILs

To identify conserved protein motifs, the online MEME suite was used, with the discovery limited to a maximum of 3 motifs and all other parameters retaining their default settings (https://meme-suite.org/meme/tools/meme; accessed on 15 July 2025). The exon-intron architecture of each gene, including details such as number, genomic position, and length, was directly derived from the provided genome annotation file (GFF3 format). Subsequently, a comprehensive visualization integrating both gene structures and the identified motif patterns was generated using the “Visualize MEME Motif Pattern” module within TBtools (https://github.com/CJ-Chen/TBtools; accessed on 15 July 2025).

### 2.5 Chromosomal distribution and density profiling of *EgAIL* genes

The chromosomal positions of all 19 identified *EgAIL* genes were determined and subsequently visualized using the “Gene Location Visualize from GTF/GFF” function in TBtools. The *E. grandis* genome annotation file in GFF3 format was used as input. Furthermore, the corresponding gene density profile was generated via the ‘One Density Profile’ module, with a bin size set to 1 million base pairs (1 Mb) and the remaining parameters maintained at their default settings.

### 2.6 Evolutionary collinearity patterns of AIL family within *E. grandis* and across taxa

To investigate potential gene duplication events and evolutionary homology, a genome-wide scan for syntenic regions containing *EgAIL* genes was performed using TBtools. This analysis included both intragenomic collinearity within *E. grandis* and interspecies collinearity between *E. grandis* and *A. thaliana or*, *E. grandis* and *P. trichocarpa.* These analyses were performed using the ‘One Step MCScanX’ function in TBtools. Subsequently, the resulting intra-genomic collinearity were visualized using the Advanced Circos module in TBtools. The analysis was run under the following parameters: 2 CPU cores, a BLAST E-value threshold of 1e^-3^, and a maximum of 10 top BLAST hits.

### 2.7 *In silico* profiling of *cis*-regulatory elements in promoters of *EgAIL* genes

Putative promoter regions (2000 bp upstream of the translation start site [ATG]) of all 19 *EgAIL* genes were extracted using the “GFF3/GTF Sequence Extraction” tool in TBtools. These promoter sequences were analyzed for *cis-*regulatory elements using the online PlantCARE database (https://bioinformatics.psb.ugent.be/webtools/plantcare/html/; accessed 16 July 2025). To visualize the distribution of *cis*-regulatory elements across the *EgAIL* gene phylogeny, the identified *cis*-regulatory data were integrated with the phylogenetic tree for visualization using the “Basic BIOsequence View” module in TBtools.

### 2.8 Spatiotemporal and stress-responsive expression profiling of the AIL family in *E. grandis*

To examine the tissue-specific, developmental, and stress-induced expression dynamics of the *AIL family*, we retrieved expression data from our published study (Fan et al., 2024). Expression was first examined in whole one-month-old seedlings and their separated leaf, stem, and root tissues. Furthermore, expression was analyzed in roots, mature leaves, young leaves, xylem, and phloem of six-month-old plants, as well as in the 1st (stem tip), 3rd, 5th, 7th, 9th, and 11th internodes of six-month-old branches. Samples from xylem and phloem of six-year-old *Eucalyptus grandis* were also included.

Additionally, two-month-old tissue-cultured seedlings GL1 were subjected to phosphorus (P)-deficient or boron (B)-deficient treatments. Root and shoot tissues were collected at 0 (control), 6, 24, 48, 96 hours, and 21 days post-treatment. Concurrently, young leaves were collected at 0, 1, 6, 24, and 168 hours after salicylic acid (SA), methyl jasmonate (MeJA), and salt stress treatments for differential expression analysis. Salt stress was induced by irrigating with nutrient solution containing 200 mM NaCl. All treatments consisted of three biological replicates, with each replicate comprising three individual plants, to ensure reproducibility and statistical robustness.

Expression data processing and visualization were performed using the TBtools-II platform (v1.120). Raw expression values were log_2_(X + 1)-transformed and then globally normalized to enhance the visualization of differentially expressed genes with low expression levels. Hierarchical clustering was conducted using Euclidean distance and complete linkage methods, and expression heatmaps were generated across tissue types and stress time points to clearly illustrate the expression patterns and trends of the *AIL family*.

### 2.9 Tertiary structure modeling and alignment of AIL family proteins in *E. grandis*

To characterize the tertiary structural features of proteins encoded by genes within the five clades of the phylogenetic tree, two genes were selected from each clade for tertiary structure alignment.

The AlphaFold-predicted tertiary structure files of the target proteins were retrieved from the UniProt database (https://www.uniprot.org/uniprotkb), followed by uploading to the Dail server (http://ekhidna2.biocenter.helsinki.fi/dali/) for pairwise structural alignment. For each clade, the gene pair with the smallest root-mean-square deviation (RMSD; a lower RMSD value indicates higher structural similarity) was selected.

Subsequently, 3D structural alignment was then verified and visualized in PyMOL 2.5.4 using the “align” command, and the structural superposition was visualized to validate the results. These analyses provided structural evidence supporting the rationality of phylogenetic tree clustering and the functional associations among genes.

## 3 Results

### 3.1 Identification and physicochemical properties analysis of the AIL family in *E.grandis*

**Figure 1:**
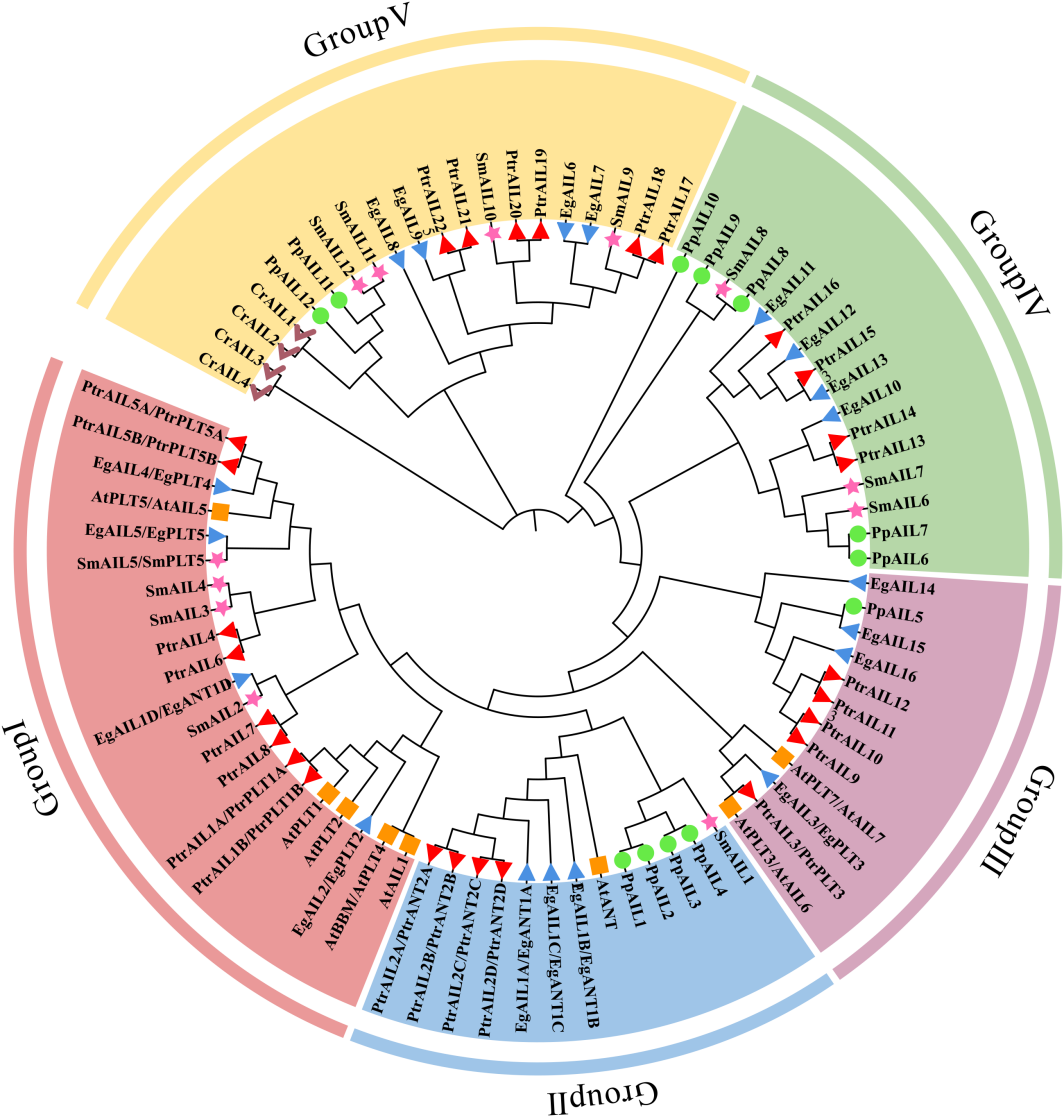
Phylogenetic reconstruction and evolutionary diversification of AIL proteins across representative species. Members of the AIL family were identified across five evolutionary lineages, with the following counts: E. grandis (Eg, 19 genes, blue, triangles), P. trichocarpa (Ptr, 27 genes, red, inverted triangles), A. thaliana (At, 8 genes, orange, squares), S. moellendorffii (Sm, 12 genes, pink, asterisks), P. patens (Pp, 12 genes, green, circles), and C. reinhardtii (Cr, 4 genes, purple, hooks). Phylogenetic relationships were inferred using the neighbor-joining method with complete AIL sequences analyzed via MEGA11 software and validated by 1000 bootstrap replicates. All AIL proteins in the figure are classified into five subfamilies, labeled in red, blue, purple, green, and yellow, respectively.

Genome-wide screening using BLASTp identified 19 putative members of the AIL family within the *E. grandis* genome. Following the established nomenclature scheme for *A. thaliana*, these genes were systematically named from *EgAIL1A*/*EgPLT1A* to *EgAIL16* (**Figure 1**; **Table 1**, **Supplementary Table 1**). In silico analysis of the deduced proteins revealed a considerable variation in length, ranging from 309 (*EgAIL15*) to 679 (*EgAIL5*/*EgPLT5*) amino acid residues. Their predicted molecular weights were distributed between 33.97 and 74.04 kDa. Similarly, the isoelectric points (pI) covered a broad spectrum from 5.67 to 9.38, thereby classifying 14 members as acidic (pI < 7) and 5 as basic (pI > 7). The instability index (II) was calculated to be between 44.43 and 64.43. Notably, all members displayed II values above 40, suggesting that the majority of *EgAIL* proteins are inherently unstable. The aliphatic index varied from 51.82 (*EgAIL1D*/*EgPLT1D*) to 70.26 (*EgAIL12*). Furthermore, the consistently negative Grand Average of Hydropathicity (GRAVY) values across all members confirmed their overall hydrophilic characteristics.

**Table 1:** *In silico*-derived physicochemical parameters of the AIL family members in *E. grandis*.

Name	Gene ID	Number of amino acids (aa)	Molecular weight (Da)	Theoretical (pI)	Instability index	Aliphatic index	Grand average of hydropathicity
*EgAIL1A/EgPLT1A*	Eucgr.F02223.1.v2.0	662	72.71	6.93	61.9	59.41	-0.682
*EgAIL1B/EgPLT1B*	Eucgr.F00098.1.v2.0	540	59.44	8.04	49.56	67.56	-0.568
*EgAIL1C/EgPLT1C*	Eucgr.H02335.1.v2.0	464	51.95	8.3	51.22	60.02	-0.744
*EgAIL1D/EgPLT1D*	Eucgr.F04421.1.v2.0	611	66.09	5.99	47.23	51.82	-0.766
*EgAIL2/EgPLT2*	Eucgr.J00792.1.v2.0	547	59.84	6.35	49.45	57.64	-0.724
*EgAIL3/EgPLT3*	Eucgr.I00564.1.v2.0	503	54.45	6.58	50.48	63.2	-0.505
*EgAIL4/EgPLT4*	Eucgr.C02333.1.v2.0	536	58.42	6.68	50.3	51.83	-0.788
*EgAIL5/EgPLT5*	Eucgr.B01460.1.v2.0	679	74.04	6.19	46.66	59.99	-0.688
*EgAIL6*	Eucgr.B02453.1.v2.0	458	49.95	5.67	49.55	54.37	-0.628
*EgAIL7*	Eucgr.J02113.1.v2.0	483	52.81	5.69	49.76	63.89	-0.543
*EgAIL8*	Eucgr.G02793.1.v2.0	488	53.03	8.11	60.43	57.68	-0.685
*EgAIL9*	Eucgr.I00892.1.v2.0	553	60.92	6.13	62.88	52.44	-0.868
*EgAIL10*	Eucgr.J00316.1.v2.0	412	45.64	5.72	64.43	62.01	-0.74
*EgAIL11*	Eucgr.B03412.1.v2.0	356	39.93	9.38	61.11	64.97	-0.714
*EgAIL12*	Eucgr.J02131.1.v2.0	389	44.07	8.19	61.69	70.26	-0.632
*EgAIL13*	Eucgr.I01921.1.v2.0	402	45.51	6.78	55.85	68.21	-0.681
*EgAIL14*	Eucgr.C00238.1.v2.0	335	38.25	6.77	47.54	57.97	-0.904
*EgAIL15*	Eucgr.C02520.1.v2.0	309	33.97	4.92	44.43	68.35	-0.446
*EgAIL16*	Eucgr.F03987.1.v2.0	349	38.14	6.1	55.12	56.1	-0.797

Collectively, these in silico analyses indicate that the 19 EgAIL proteins exhibit common physicochemical characteristics: significant variation in amino acid length, predominantly acidic nature, overall low stability, and hydrophilicity.

### 3.2 Phylogenetic analysis of EgAIL proteins

Phylogenetic analysis revealed that the 19 EgAIL family members can be grouped into five phylogenetic clusters (Group I–V), comprising 4, 3, 4, 4, and 4 members respectively (**Figure 1**, **Supplementary data 2**). Overall, *EgAIL* genes are relatively evenly distributed across these clusters, with Groups I, III, IV, and V each containing four members, while Group II has three members. No *A. thaliana AIL* gene was detected in Groups IV and V, while all four *AIL* members from *C. reinhardtii* were clustered within Group V. Phylogenetic analysis indicated that most EgAILs share the closest evolutionary relationship with PtrAILs from *P. trichocarpa*, followed by *A. thaliana*. Specifically, *EgAIL2* clustered with *AtPLT2*, *EgAIL3* with *AtPLT3/AtAIL6*, and *EgAIL5* with *AtPLT5/AtAIL5*, suggesting these members may share conserved functional characteristics. Phylogenetic relationships between *E. grandis* and *S. moellendorffii*, *P. patens*, and *C. reinhardtii* progressively weakened. Collectively, these results revealed that the *E. grandis* AIL family exhibited highest homology with the orthologous genes of the woody plant *P. trichocarpa*, followed by the dicotyledonous *A. thaliana*. As the evolutionary rank decreased from angiosperms to ferns, mosses, and algae, the evolutionary distance between *EgAIL* genes and their homologous AIL genes progressively increased. This distribution pattern indicated that the functional differentiation of the AIL genes correlated with their phylogenetic position. Species with closer evolutionary relationships exhibited higher evolutionary conservation in their homologous genes, consistent with the typical characteristics of gene family evolution.

### 3.3 Gene structure and conserved motif analyses of EgAILs

**Figure 2:**
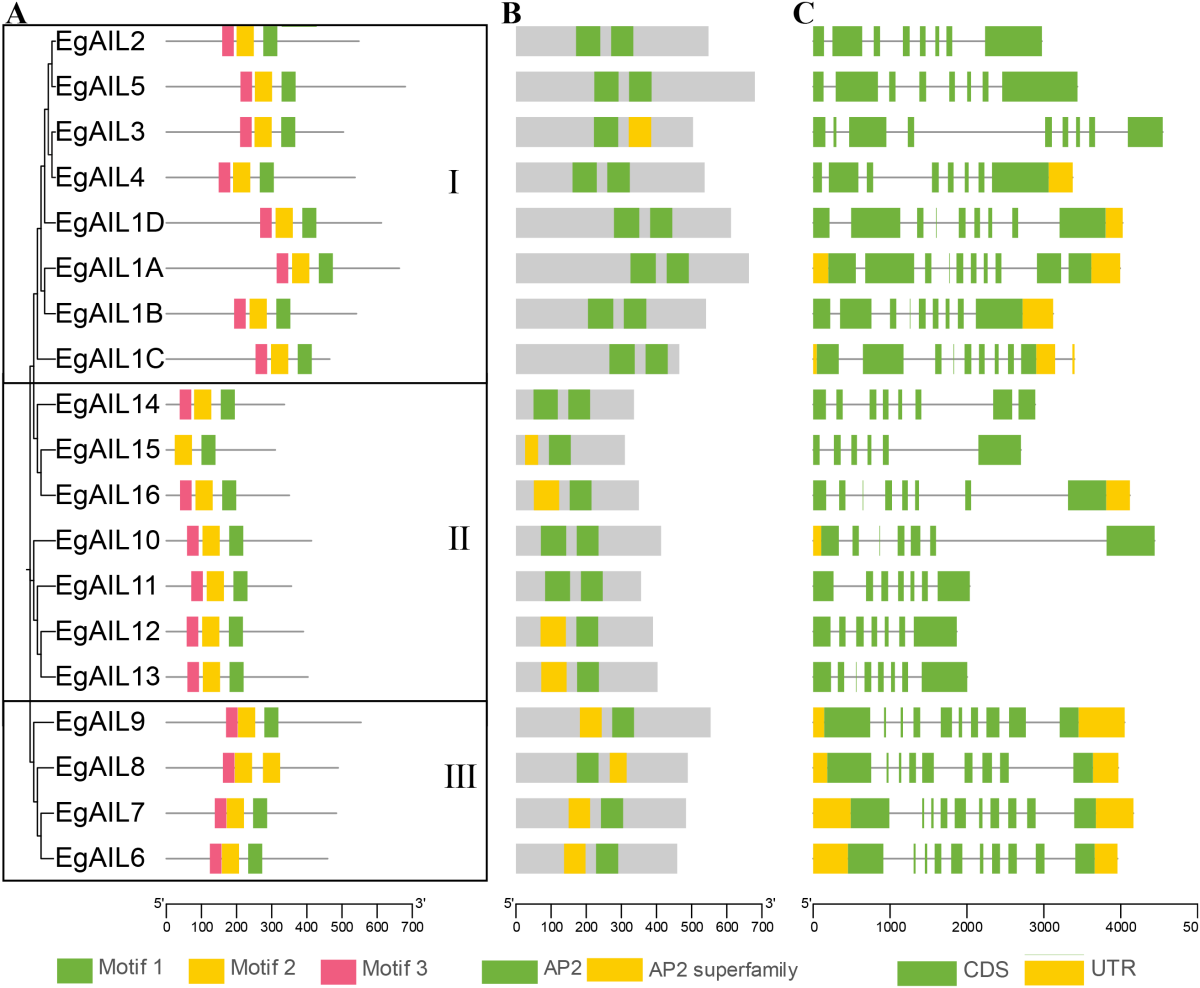
Conserved motif patterns and gene architectures in the context of AIL protein phylogeny in Eucalyptus grandis. **(A)** Phylogenetic reconstruction of EgAIL proteins using maximum likelihood methodology delineates evolutionary subfamilies, with corresponding color-coded distributions of three conserved motifs (1-3). **(B)** Architectural organization of two conserved AP2 domains within EgAIL proteins. **(C)** Genomic structure of EgAIL proteins illustrating exon-intron organization. Scale bars in kb, kilobases are provided for all panels.

Based on genomic annotation data from *E. grandis*, the MEME system was employed to analyze the conserved motifs and gene structures of 19 *EgAIL* genes. Based on the understanding of conserved features within the AIL family from previous studies (Liu et al., 2023), we identified three conserved protein motifs (Motifs 1–3) (**Figure 2A**). From a phylogenetic perspective (**Figure 2A**), all members except *EgAIL15* (Group II) contained motifs 1-3, with similar compositional orders, indicating high conservation of motifs 1–3 and suggesting that *EgAIL15* may have lost part of its structure during replication. All members contained two classic AP2 domains (**Figure 2B**), confirming their classification within the AIL family. Finally, analysis of coding sequences (CDS) and untranslated regions (UTR) (**Figure 2C**) revealed significant heterogeneity among members in exon-intron structure, intron size, and intron number, highlighting substantial structural diversity within this gene family.

### 3.4 Chromosome distribution of *EgAIL* genes

**Figure 3:**
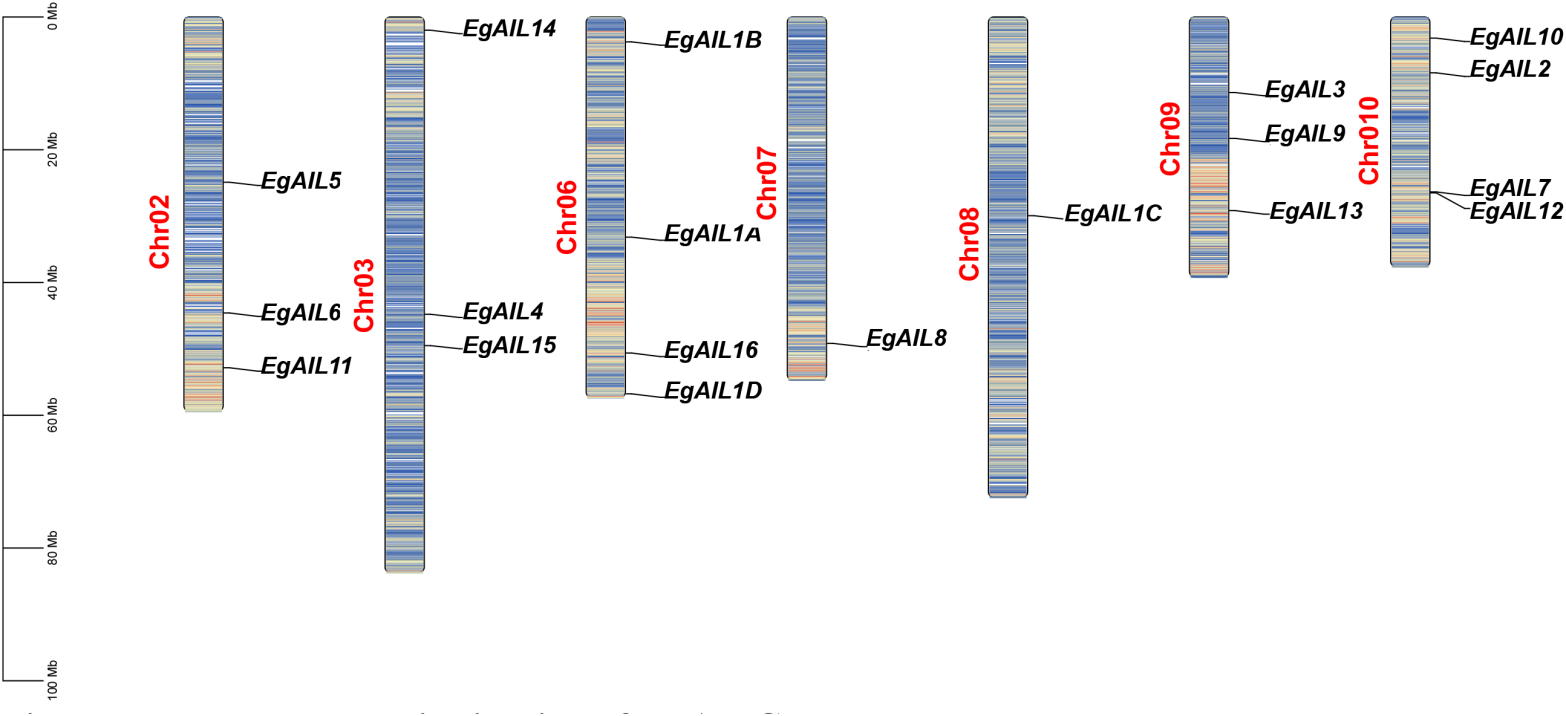
Chromosomal distribution of EgAIL genes. A scale bar at the left indicates physical distances in mb, megabases. Chromosomal ideograms adjacent to the scale use red and blue segments to represent regions of high and low gene density, respectively. Black annotations to the left of each ideogram identify chromosome numbers, while corresponding AIL gene names appear in black on the right. Guiding lines connect each gene to its specific cytogenetic location.

Based on genome annotation information for *E. grandis*, chromosomal localization analysis was conducted on the 19 *EgAIL* genes. The 19 *EgAIL* genes exhibited a dispersed arrangement across seven chromosomes (**Figure 3**). There were significant differences in the number of *EgAIL* genes carried by each chromosome, with Chr06 and Chr10 harboring the largest number of members (n = 4) and Chr07 and Chr08 harboring only one member (n = 1). Overall, the *EgAIL* genes exhibited dispersed distributions on the genome, thereby providing valuable genomic insights into the mechanisms of gene family expansion and potential functional coordination among their members.

### 3.5 Collinearity analysis of the *EgAIL* genes

**Figure 4:**
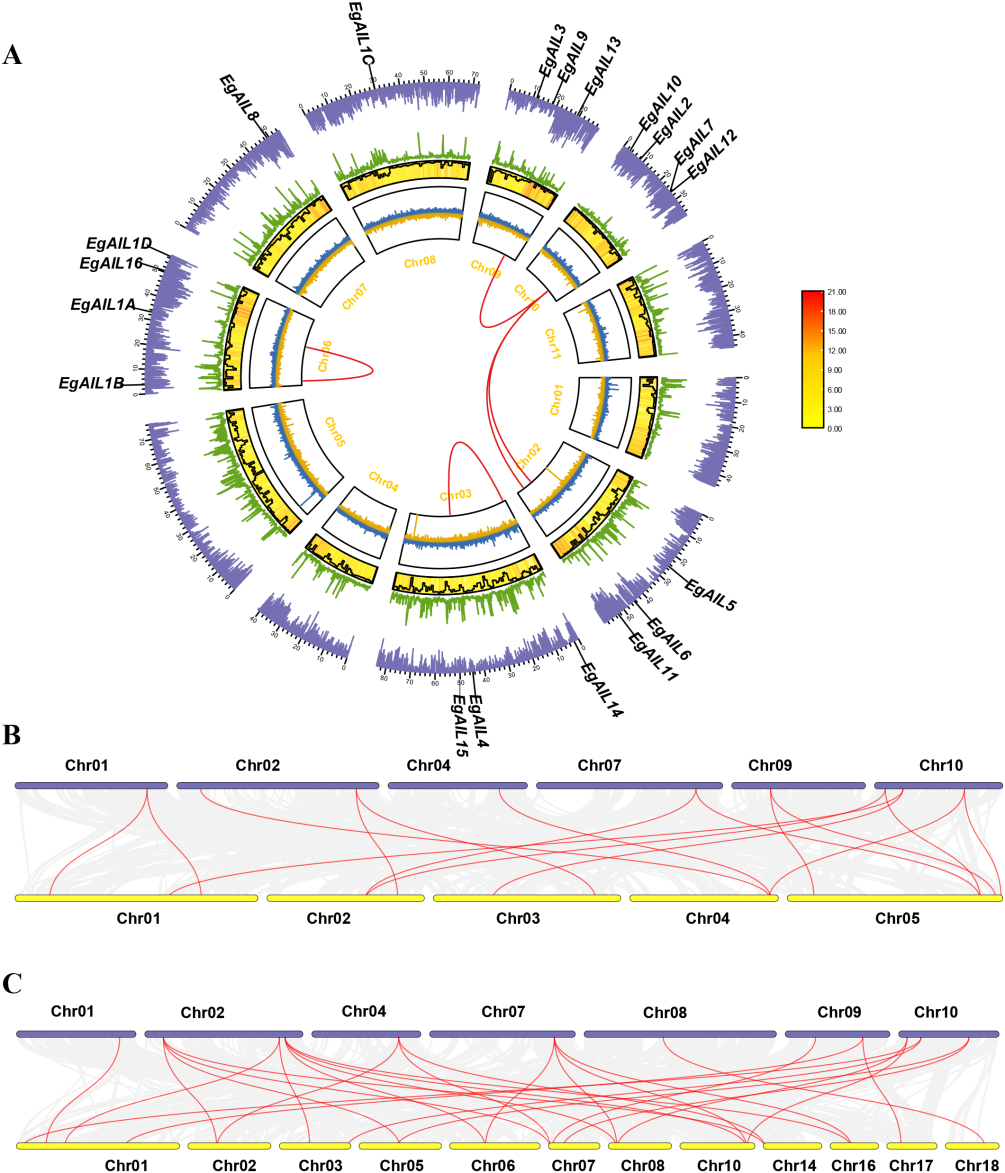
Comparative collinearity analysis of the AIL family among (E) grandis, (A) thaliana, and (P) trichocarpa. **(A)** Intra-genomic collinearity within (E) grandis. Gray lines represent overall syntenic blocks; red lines highlight segmental duplication events involving EgAIL genes. **(B)** Inter-specific collinearity between (E) grandis and (A) thaliana. **(C)** Comparative collinearity between (E) grandis and (P) trichocarpa. In panels **(B, C)**, gray lines indicate conserved syntenic regions between species, while red lines denote orthologous relationships of AIL genes.

To investigate the genomic duplication mechanisms underpinning the expansion of the AIL family, an intra-genomic collinearity analysis was performed in the *E. grandis* genome (**Figure 4A**). Tandem and segmental duplications were established as fundamental evolutionary forces that drive gene family diversification and facilitate adaptive evolution (Panchy et al., 2016). The analysis identified five paralogous pairs, being indicative of both tandem and segmental duplication events. Specifically, these pairings included *EgAIL1B*–*EgAIL1A*, *EgAIL14*–*EgAIL15*, *EgAIL12*, and *EgAIL7* respectively paired with *EgAIL6*, *EgAIL11*, and *EgAIL13*.

Inter-species collinearity analysis identified 16 orthologous AIL pairs between *A. thaliana* and *E. grandis*, whereas 30 such syntenic pairs were detected between *E. grandis* and *P. trichocarpa* (**Figures 4B, C**). The nearly two-fold higher number of syntenic pairs with *P. trichocarpa* reflected a closer evolutionary and phylogenetic relationship in *AIL* gene organization.

### 3.6 Cis-regulatory element analysis of *EgAIL* genes

**Figure 5:**
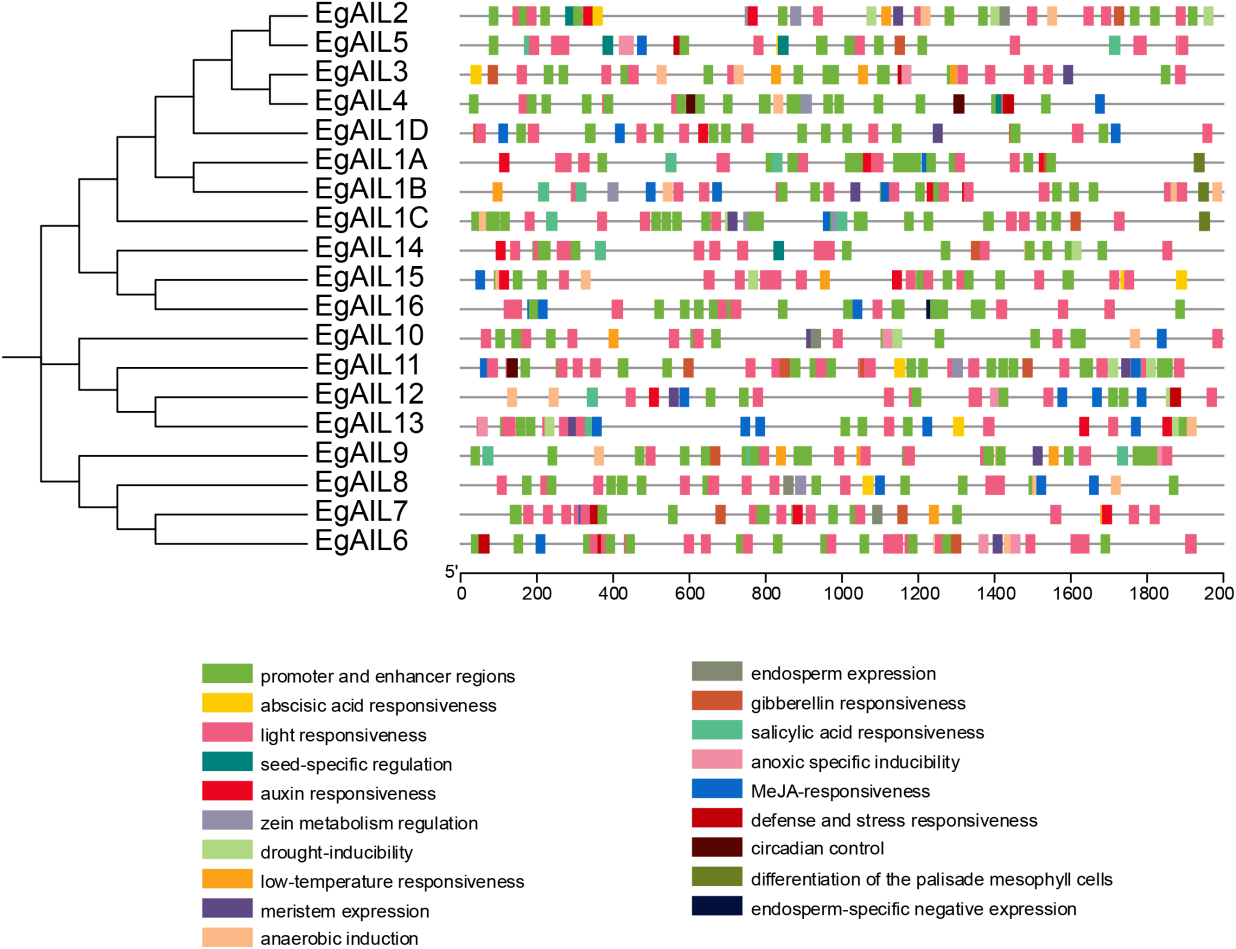
Distribution and functional diversity of Cis-acting elements in the promoters of EgAIL genes. The diagram displays the 2000 bp upstream promoter regions of all EgAIL genes, with colored modules representing distinct cis-regulatory elements. A full legend of element annotations is provided below the plot.

Leveraging the *E. grandis* genome annotation, a systematic analysis for *cis-*regulatory elements was conducted within the 2 kb promoter regions upstream of the translational start sites for all 19 *EgAIL* genes. The distribution of these elements was subsequently analyzed in the context of the established phylogenetic framework (**Figure 5**). Gene transcriptional regulation is strongly dependent on the types, copy numbers, and spatial distribution of *cis-*elements within the promoter regions, as well as their specific binding to cognate transcription factors (TFs) (Wang et al., 2014). Systematic profiling identified 19 distinct *cis*-regulatory elements in the promoter regions of all 19 *EgAIL* genes. These elements were systematically classified into four major functional categories (Supplementary Data 3): hormone-responsive (5 types), light-responsive (1 type), abiotic stress-responsive (5 types), and development-related (8 types). Statistical results indicated that among *cis*-acting elements across different functional categories, promoter and enhancer regions account for the highest number of elements (236), followed by light-responsive elements (212). Furthermore, development-related elements exhibited the greatest diversity, encompassing eight distinct functional subcategories related to various tissue and physiological processes, including endosperm expression and seed-specific regulation. The majority of development-related elements were linked to meristem development, a finding consistent with the established role of AIL in somatic embryogenesis and tissue differentiation. Light-responsive elements were present across all family members, suggesting that light signals are a key environmental factor regulating *AIL* transcription and, through downstream networks, influencing plant growth and development. Elements responsive to MeJA and gibberellins (GAs) were also prevalent, indicating that *EgAIL* expression is modulated by these hormones and suggesting their involvement in hormonal signaling pathways that integrate growth, development, and environmental responses. Furthermore, the *EgAIL16* promoter was found to contain a unique endodermis-specific negative regulatory element.

### 3.7 Tissue- and development-specific expression pattern analysis of *EgAIL* genes

**Figure 6:**
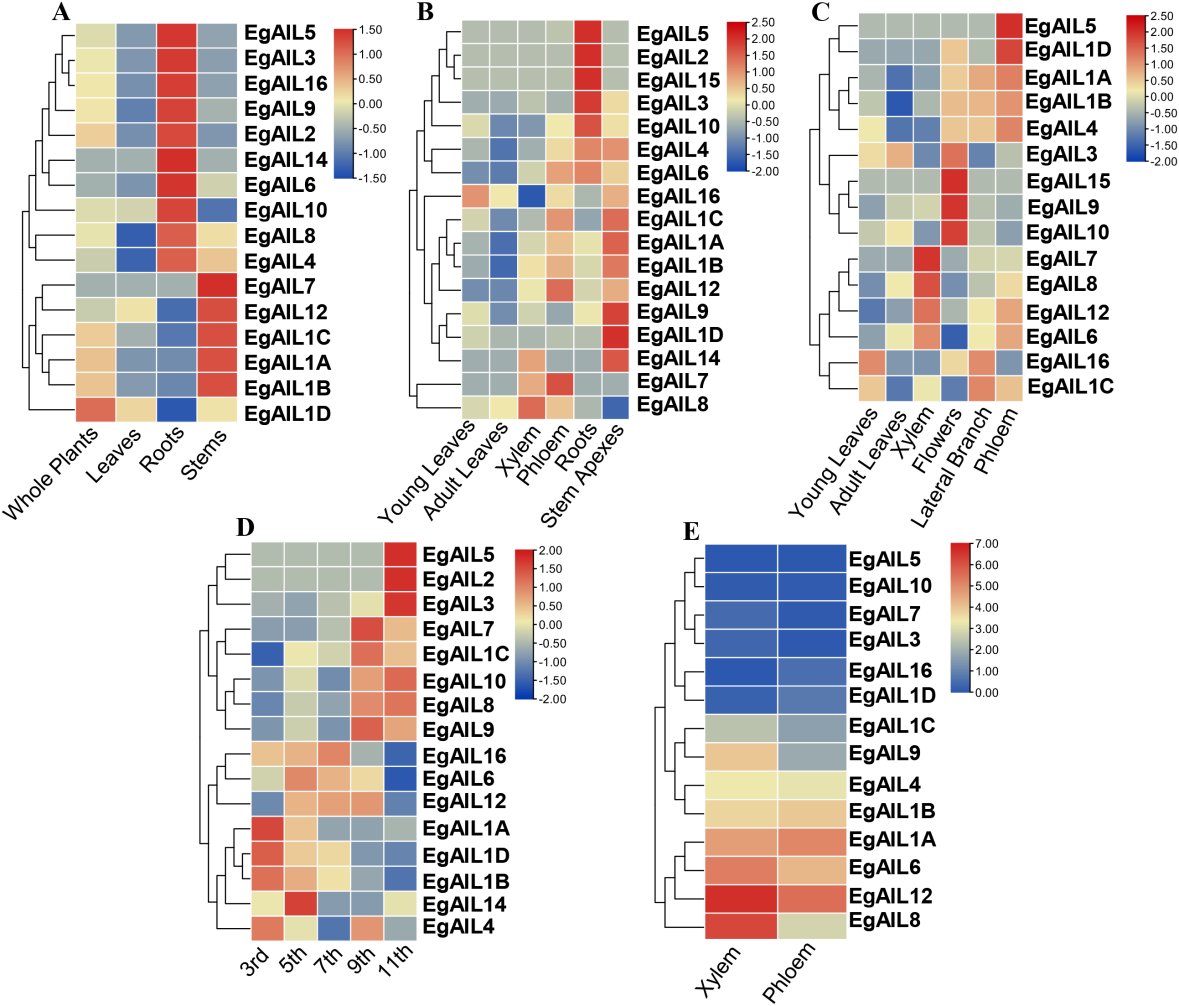
Tissue-specific expression profiles of EgAIL genes. Expression patterns are shown across diverse developmental stages and organs: **(A)** whole plants, leaves, roots, and stems of 30-day-old tissue cultured plants; **(B)** young leaves, adult leaves, xylem, phloem, roots, and stem apexes of 6-month-old plants; **(C)** young leaves, adult leaves, xylem, flowers, lateral branches and phloem of 3-year-old plants; **(D)** stem internodes sampled from 6-month-old shoots included the 3rd, 5th, 7th, 9th, and 11th internodes; **(E)** xylem and phloem of 6-year-old trees. Expression levels are represented by a color gradient from blue (low) to red (high).

Subsequently, we performed a characterization of the tissue- and development-specific expression profiles of the EgAIL family in *E. grandis*, based on the published dataset (Fan et al., 2024). Expression analysis was conducted on 17 out of the 19 *EgAIL* family members (*EgAIL11* and *EgAIL13* were excluded due to the absence of transcriptomic data) across a range of tissue samples. The results revealed distinct tissue-specific and developmental stage-specific expression patterns (**Figure 6**).

For example, in 30-day-old tissue-cultured seedlings, *EgAIL2*, *EgAIL3*, *EgAIL4, EgAIL5*, *EgAIL6*, *EgAIL8*, *EgAIL9*, *EgAIL10*, *EgAIL14* and *EgAIL16* showed the highest expression levels in roots, whereas *EgAIL1A*, *EgAIL1B*, *EgAIL1C*, *EgAIL7* and *EgAIL12* were most highly expressed in stems. Notably, only *EgAIL1D* was significantly expressed throughout whole plants. (**Figure 6A**). In 6-month-old plants, *EgAIL2*, *EgAIL3, EgAIL4, EgAIL5*, *EgAIL6*, *EgAIL10*, and *EgAIL15* maintained this root-preferential expression pattern, while *EgAIL1A*, *EgAIL1B*, *EgAIL1C*, *EgAIL1D, EgAIL9* and *EgAIL14* exhibited the highest expression in stem apices (**Figure 6B**). In 3-year-old plants, *EgAIL1A*, *EgAIL1B*, *EgAIL1D*, *EgAIL4* and *EgAIL5* were most highly expressed in phloem. *EgAIL7*, *EgAIL8*, *EgAIL6*, and *EgAIL12* showed elevated expression in the xylem, while *EgAIL3*, *EgAIL9*, *EgAIL10* and *EgAIL15* showed peak expression in flowers (**Figure 6C**). In stem internodes collected from 6-month-old plants, some *EgAIL* genes displayed strong expression in apical internodes (e.g., *EgAIL1A*, *EgAIL1B*, *EgAIL1D* and *EgAIL4*), whereas others were more highly expressed in basal internodes (e.g., *EgAIL2*, *EgAIL3* and *EgAIL5*) (**Figure 6D**). In both xylem and phloem samples, *EgAIL5*, *EgAIL10*, *EgAIL7*, *EgAIL3*, *EgAIL16*, and *EgAIL1D* exhibited low expression levels. By contrast, *EgAIL1A*, *EgAIL6*, *EgAIL12*, and *EgAIL8* were strongly expressed in the xylem, with *EgAIL1A* and *EgAIL12* also showing high expression in the phloem (**Figure 6E**).

### 3.8 Expression of the *EgAILs* upon abiotic stress and plant hormone treatments

**Figure 7:**
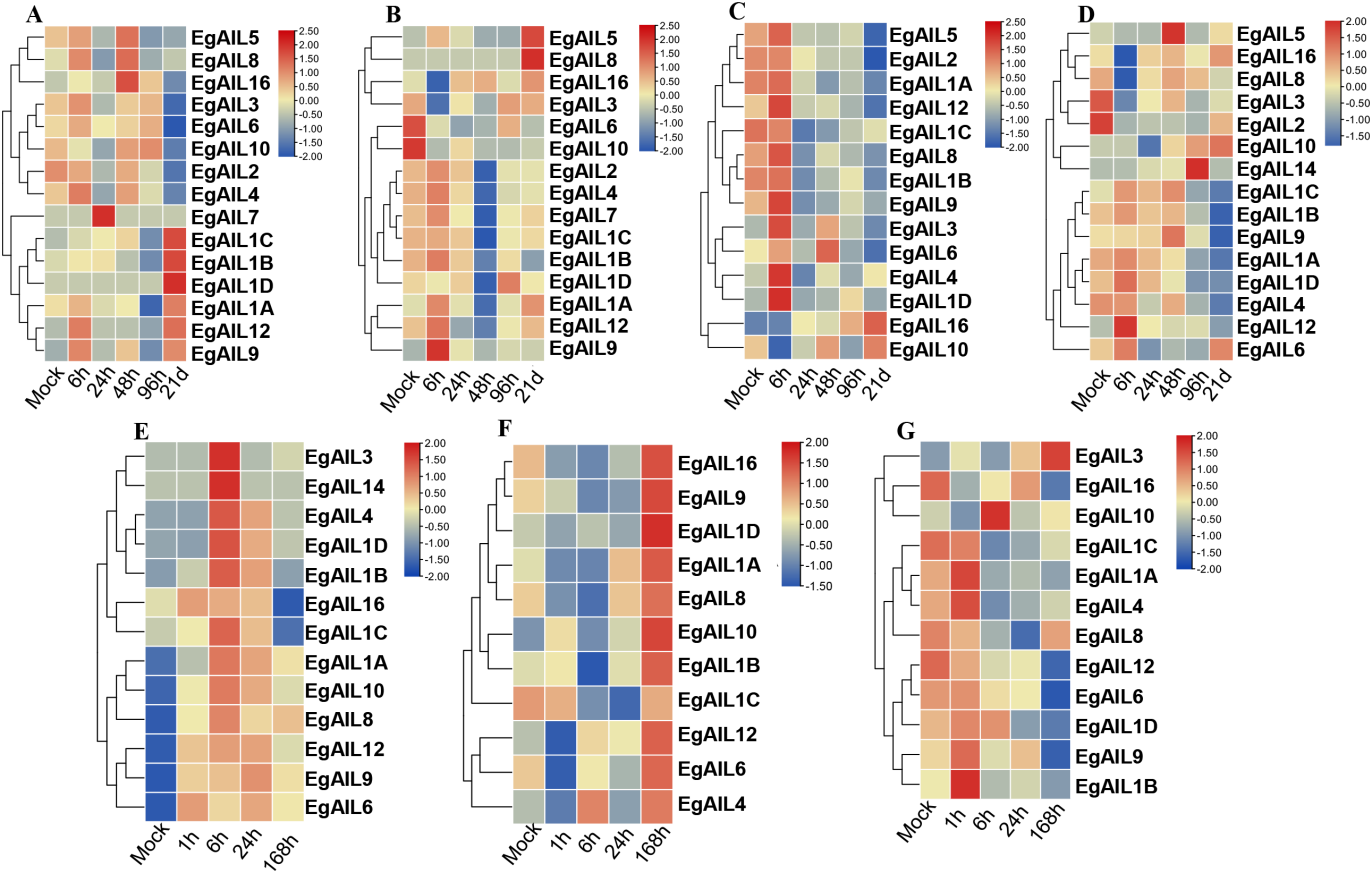
Expression profiles of EgAILs under abiotic stress and phytohormone treatments. **(A, B)** Temporal expression profiles in root **(A)** and shoot **(B)** tissues during a phosphorus deficiency time-course (0, 6, 24, 48, 96 hours; 21 days). **(C, D)** Transcriptional dynamics in root **(C)** and shoot **(D)** tissues in response to boron deficiency, sampled at identical intervals. **(E–G)** Expression patterns in young leaves following treatment with MeJA, methyl jasmonate; **(E)**, SA, salicylic acid; **(F)**, and salt stress **(G)**, assessed at 0, 1, 6, 24, and 168 hours post-elicitation.

To characterize the transcriptional dynamics of the EgAIL family in response to abiotic stress and phytohormone signaling, two-month-old *E. grandis* plants were subjected to a series of treatments including phosphorus deficiency, boron deficiency, salt stress, and foliar sprays of SA and MeJA (Fan et al., 2024). The *cis*-acting element mapping revealed that the promoters of *EgAIL* genes contain *cis-*elements associated with MeJA, SA, and abiotic stress (**Figure 5**), thus suggesting a regulatory basis for the gene family’s response to these signals. However, transcriptomic data revealed significant temporal and tissue-specific expression patterns, indicating that the function of *cis*-elements is not determined by individual elements alone but is regulated by their combinations, including copy numbers, spatial arrangements, and specific interactions with transcription factors. Under short-term phosphorus deficiency treatment (6–96 h), expression in roots of most *EgAIL*s showed no significant changes. However, *EgAIL7* exhibited markedly elevated expression at 24 h post-treatment, while *EgAIL16* reached peak expression at 48 h. Under the 21-day long-term treatment, *EgAIL1A*, *EgAIL1B*, *EgAIL1C*, *EgAIL1D*, *EgAIL12* and *EgAIL9* showed significant expression, while *EgAIL2*, *EgAIL3*, *EgAIL4*, *EgAIL6* and *EgAIL10* exhibited significantly reduced expression. (**Figure 7A**). In contrast, in young shoots, the vast majority of *EgAIL* genes showed significant downregulation under short-term treatment (**Figure 7B**). Exceptions included *EgAIL12*, which reached peak expression at 6 h post-treatment, and *EgAIL5* and *EgAIL14*, which exhibited minimal change during short-term treatment but showed significant upregulation only after 21 d of long-term treatment. (**Figure 7B**). Under boron deficiency conditions, root tissues exhibited a pattern where most *EgAILs* gene expression initially increased and then decreased following short-term treatments lasting 6–96 h, while *EgAIL16* expression showed a gradual increase trend. After 21 d of long-term treatment, expression of all genes except *EgAIL1C*, *EgAIL4*, *EgAIL10* and *EgAIL16* was downregulated. (**Figure 7C**). In young shoots, *EgAIL5*, *EgAIL10* and *EgAIL14* were markedly induced, while *EgAIL1A*, *EgAIL1B*, *EgAIL1C*, *EgAIL1D*, *EgAIL4*, *EgAIL9*, and *EgAIL12* were repressed (**Figure 7D**).

Under hormonal treatment, MeJA application induced high expression of all *EgAIL* genes in young leaves at 6 h post-treatment, followed by a subsequent decline in expression. Among these, *EgAIL16* and *EgAIL1C* showed significant downregulation at 168 h post-treatment. (**Figure 7E**). All of the detected *EgAIL* genes except *EgAIL1C* showed upregulated expression after SA treatment (**Figure 7F**). Under salt stress, young leaves exhibited upregulation of *EgAIL3*, whereas other genes showed reduced expression or a pattern of initial induction followed by reduction (**Figure 7G**).

## 4 Discussion

Transcription factors function as master regulators that integrate diverse signaling pathways and play key roles in plant growth, development, and environmental adaptation (Strader et al., 2022). *AIL* genes, which encode a class of core plant-specific transcription factors, have been extensively characterized in model plants like *A. thaliana*, where they play critical roles in somatic embryogenesis and the maintenance of stem cell homeostasis (Li et al., 2021). Previous research has established that *AIL* genes are ubiquitously distributed across higher plant lineages, where they serve as master regulators governing somatic embryogenesis and organ regeneration (Liu et al., 2023). Given these fundamental roles, they are recognized as major biotechnological targets for enhancing plant propagation efficiency (Skinner et al., 2023; Shi and Ma, 2025), improving genetic transformation, and increasing stress adaptability. Therefore, characterizing *AIL* genes and their orthologs in economically important woody plants holds great promise for advancing agricultural biotechnology and accelerating molecular breeding programs.

This study identified 19 *AIL* genes in the *E. grandis* genome, and they were designated *EgAIL1A* to *EgAIL16* following a systematic nomenclature. Physicochemical characterization revealed substantial diversity among these members, including wide variations in length, a predominance of acidic isoelectric points, relatively low stability, and overall hydrophilic character (**Table 1**). Phylogenetic analysis indicated that the evolutionary relationships of *EgAIL* genes followed a linear progression from lower to higher plant lineages (**Figure 1**). Most *EgAILs* exhibited closer affinity with *AIL* genes from *P. trichocarpa*, followed by association with *AtAIL* genes. In contrast, a small number of *EgAIL* genes clustered with *AIL* genes from lower plants such as *S. moellendorffii* and *P. patens*. Both Group V and Group IV were rich in early-diverging plant species and they contained eight members of the *E. grandis* AIL family, despite containing no *AtAIL* genes. These findings suggest a complex evolutionary history for the AIL family in *E. grandis*, involving both the retention of ancestral sequences and lineage-specific duplication or loss events. This evolutionary dynamic likely contributed to functional diversification and sequence divergence, potentially enabling unique adaptive strategies in *E. grandis*. Compared to herbaceous model plants such as *A. thaliana*, the AIL family in *E. grandis* exhibits more pronounced expansion (19 members) and phylogenetically more diverse, as exemplified by Groups IV and V, which primarily contain homologs from early-diverging plants. A similar expansion is observed in woody plants like *P. trichocarpa* (27 members), suggesting that the amplification of the AIL family may be associated with the complex perennial development and organogenesis requirements of woody plants. This phylogeny-based functional diversification is supported by comparisons of protein tertiary structures (Supplementary Data 4). Notably, EgAIL proteins within the same phylogenetic subfamily (e.g., *EgAIL1A* and *EgAIL1B*) exhibited high structural similarity, whereas representatives from distinct subfamilies (e.g., *EgAIL5* and *EgAIL*8) showed marked structural divergence. This congruence between phylogenetic grouping and protein structural conservation underscores that the evolutionary history of the EgAIL family has likely given rise to functionally distinct protein clades.

Furthermore, the close evolutionary relationships of *EgAIL1A*, *EgAIL1B*, and *EgAIL1C* with *AtANT* suggest that these genes may play roles in floral organ development, possibly involving functional overlap with *ANT* (Kim et al., 2006; Krizek et al., 2023) (**Figure 1**). Although this study provides fundamental insights into the functional diversification of the AIL family in *E. grandis*, the precise molecular mechanisms and functional divergence among *EgAIL* paralogs require further experimental validation.

Collinearity analysis provided further insights into the evolutionary trajectory of the family. Intraspecific collinearity analysis revealed 5 pairs of genes, some of which had collinear regions with genes outside the family (**Figure 4A**). Interspecific collinearity assessments identified markedly stronger genomic collinearity between *E. grandis* and *P. trichocarpa* compared to *A. thaliana*, reflecting their shared phylogenetic affinity as woody plants (**Figures 4B, C**). Collectively, these findings suggest that the EgAIL family has expanded primarily via localized duplication events while maintaining core functions in somatic embryogenesis and cell proliferation. The larger size of the EgAIL family compared to that of *A. thaliana* implies that gene duplication and subsequent functional diversification have increased the complexity of its regulatory networks, thereby enhancing the adaptive capacity for growth, development, and environmental stress response in *E. grandis*.

*Cis*-regulatory elements (CREs) act as pivotal regulatory components in orchestrating the transcriptional programs that govern plant growth and development. Consistent with this principle, our promoter analysis has disclosed that *EgAIL* genes contain an abundance of *cis-*elements related to development and light-responsiveness (**Figure 5**). The presence of these elements, along with those responsive to MeJA and GAs, suggests that *EgAIL* genes likely integrate endogenous hormonal signaling pathways with external photoperiod signals to regulate somatic embryogenesis and organ regeneration. Such a complex regulatory architecture likely contributes to an important molecular basis of efficient clonal propagation in *E. grandis* and may also underpin its ecological adaptability.

The expression pattern of the *EgAIL* genes strongly support the core role of the AIL family in meristem activity and organogenesis, while also revealing potential characteristics of *E. grandis* as a fast-growing forest tree. Members of the *EgAIL family* were highly expressed in young tissues and actively dividing regions across multiple developmental stages (**Figure 6**). Specifically, *EgAIL1A*, *EgAIL1B*, and *EgAIL1C* were highly expressed in the shoot apices of both young and mature *E. grandis* (**Figures 6A, B**). Phylogenetically, these genes clustered with the *A. thaliana* homolog *ANT*. In *A. thaliana*, *ANT*, *AIL6/PLT3*, and *AIL7/PLT7*​ are known to synergistically maintain the continuous function of the shoot apical meristem (SAM) through unique and non-redundant roles, and their loss leads to premature SAM termination and altered expression of key regulators like WUS and STM (Mudunkothge and Krizek, 2012). Thus, the sustained high expression pattern of *EgAIL1A*, *EgAIL1B*, and *EgAIL1C* from seedlings to mature tree apices suggests they may form a functionally redundant or synergistic transcriptional module dedicated to maintaining the long-term activity and continuous organ-producing capacity of the *E. grandis* apical meristem.

A distinct, root-specific expression pattern was observed for another set of genes. *EgAIL2*, *EgAIL3*, *EgAIL5*, and *EgAIL10* were consistently highly expressed in root tissues of both 30-day-old seedlings and 6-month-old plants. Phylogenetic analysis provided insights into their putative functions: *EgAIL2* and *EgAIL5* clustered with the key root development genes *PLT1* and *PLT2* in *A. thaliana*, while *EgAIL3* grouped with *PLT3*, which is specifically expressed in root stem cells. In *A. thaliana*, *PLT1* and *PLT2* were core factors for root stem cell niche specification and maintenance, depending on auxin signaling (Passarinho et al., 2008; Rigal et al., 2012), and *PLT3* plays a specialized role in maintaining root stem cell activity (Aida et al., 2004; Galinha et al., 2007). The co-expression of these *EgAILs*, coupled with their phylogenetic affinity to these well-characterized *A. thaliana* genes, strongly suggests they may function as a conserved “root development module” in *E. grandis*, likely involved in stem cell maintenance, meristem activity, and root architecture establishment. Notably, the inclusion of the phylogenetically distant *EgAIL10* in this expression module may indicate a species-specific evolutionary expansion, potentially linked to the need for forming larger, more complex root structures to support fast growth.

Beyond the apical and root meristems, the expression profile of *EgAIL* genes suggests involvement in broader developmental contexts. Unlike their *A. thaliana* homologs, which are primarily expressed in root tips, several *EgAILs* (such as *EgAIL1A*, *EgAIL12*, and *EgAIL6*) showed significant expression in the secondary xylem and phloem of mature trees. This implies that in forest trees, AIL family genes may participate in the regulation of secondary growth, representing a promising new direction for investigation. Furthermore, *EgAIL16*​ was consistently​ expressed in young and immature leaves (**Figure 6B**), suggesting a role in early leaf development. It is noteworthy that *EgAIL16* is also predicted to contain a unique endodermis-specific​ negative regulatory element, reminiscent of the spatiotemporal-specific expression and negative regulation mechanisms employed by AIL/PLT family in *A. thaliana* to fine-tune cell proliferation and prevent over-proliferation (Horstman et al., 2015). The high transcriptional abundance of *EgAIL* genes across root, stem, and floral organ systems aligns with their canonical functions in promoting organ morphogenesis and regulating embryogenic pathways. The putative functional modules (e.g., shoot apex-associated and root-specific groups) inferred from these expression patterns are further supported by protein tertiary structure analysis, which shows high conservation within modules and divergence between them, providing a structural basis for functional specialization.

The EgAIL family exhibits extensive and differentiated responses to multiple hormones and abiotic stresses, suggesting its potential role as a key node integrating developmental and stress signaling pathways. Transcriptome-wide expression analysis under a range of abiotic and hormonal stimuli highlights the pivotal roles of MeJA and SA in regulating the expression of the EgAIL family, consistent with the hormone-responsive nature of AIL family genes in plants. As a key signaling molecule, MeJA orchestrated a range of physiological processes, from mediating responses to biotic and abiotic stress to regulating seed germination, organogenesis, and flowering (Wu et al., 2010; Ruan et al., 2019; Li et al., 2022). Previous studies showed that MeJA helped maintain ionic homeostasis in maize (Mir et al., 2018) and acted as a critical regulator in plant stress signaling pathways (Piotrowska et al., 2009). SA, in conjunction with antagonistic biocontrol bacteria, has been shown to significantly improve control of *E. grandis* bacterial wilt (Ran et al., 2008). Based on stress-responsive expression profiles, about two-thirds of *EgAIL* genes were upregulated under prolonged SA treatment (**Figure 7F**), suggesting that some family members may contribute to biotic stress defense responses. It is noteworthy that MeJA and SA can strongly induce the expression of most *EgAIL* genes, which is consistent with the analysis of their promoters being enriched with corresponding response elements (**Figure 5**). This finding aligns with previous reports on the involvement of *AIL* genes in abiotic stress responses (Kuo, 2010). However, this study is the first to systematically reveal, at the forest tree level, the strong association of the entire AIL family with these two key defense signaling pathways. This provides a new perspective for understanding how forest trees coordinate growth and defense.

In comparative analyses of phosphorus and boron deficiency, long-term boron deficiency markedly suppressed overall expression of *EgAIL* genes, most substantially in roots (**Figures 7A, C**). Long-term phosphorus deficiency, however, had a relatively limited impact, indicating that *EgAIL* genes are more sensitive to boron deficiency and that their responses may be tissue- and development-specific (Brdar-Jokanović, 2020). Under salt stress (**Figure 7G**), the vast majority of *EgAIL* genes were downregulated. This observation is consistent with the previously reported growth inhibition strategy under salinity in *Cucumis sativus* L (Yin et al., 2025), which involves *CsBBM* and *CsAIL3* and is likely a resource-conserving response. A key novel finding of this study, however, is the strong and specific induction of *EgAIL3*. Tertiary structure alignment revealed notable divergence between *EgAIL3* and other subfamily members (Supplementary Data 4), which may explain its distinct interaction partners or regulatory capacity under stress conditions. This suggests that *EgAIL3* may have evolved a unique, positive regulatory function in salt tolerance, making it a promising candidate for molecular breeding to enhance salt tolerance in *E. grandis*. Future research should focus on validating the specific role of *EgAIL3* under salt stress and elucidating its regulatory relationships with other downregulated members within the gene network.

This study employed an integrated analysis of phylogeny, protein structure, cis-regulatory elements, and expression profiles to reveal the expansion and diversification of the AIL family in *E. grandis* and to elucidate its potential roles in developmental regulation and stress responses. These findings provide multidimensional insights into the evolution and function of the *AIL* family in woody plants. It should be noted that the conclusions of this study are primarily based on bioinformatic and omics data. Further experimental validation, including qPCR analysis of key gene expression and functional characterization using genetic approaches, is required. Notably, the significant structural divergence observed among different subfamilies offers important clues for future investigation into the mechanisms underlying functional differentiation at the protein–protein interaction level. This work establishes a theoretical foundation for utilizing the AIL family in the genetic improvement of forest tree traits.

## 5 Conclusions

This research utilized an integrated genomic and transcriptomic approach combining phylogenetics, conserved domain architecture, and protein motif analyses to delineate 19 *AIL* genes in the *E. grandis* genome and categorize them into five evolutionarily distinct subfamilies. Subsequent analysis of tissue-specific and stress-responsive expression profiles established the core role of the *EgAIL* family in plant regeneration, growth, and development, with specific members inferred to be key direct factors inducing somatic embryogenesis. Furthermore, cross-species comparative analysis with *A. thaliana* and *P. trichocarpa* enabled the prioritization of candidate key regulators from the EgAIL family for future functional interrogation. In summary, this research establishes a solid genomic foundation for subsequent functional studies and offers valuable perspectives for molecular breeding initiatives, especially regarding the application of somatic embryogenesis for efficient clonal propagation of superior *E. grandis* germplasm.

## Data Availability

Publicly available datasets were analyzed in this study. The raw sequence data reported in this paper have been deposited in the Genome Sequence Archive under accession numbers PRJCA002468 and are publicly accessible at https://bigd.big.ac.cn/gsa.
