# Supplementary material for: Genome-wide identification, characterization and in-silico expression of AINTEGUMENTA-LIKE family in Eucalyptus grandis
Source: Front Plant Sci. 2026 May 1;17:1798071. doi: 10.3389/fpls.2026.1798071 (PMC13175972; doi:10.3389/fpls.2026.1798071)
Supplement: Supplementary file 2 [file DataSheet2.docx]

((((((((((((((PtrAIL5A/PtrPLT5A,PtrAIL5B/PtrPLT5B),EgAIL4/EgPLT4),AtPLT5/AtAIL5),(EgAIL5/EgPLT5,SmAIL5/SmPLT5)),((SmAIL4,SmAIL3),(PtrAIL4,PtrAIL6))),((EgAIL1D/EgANT1D,SmAIL2),(PtrAIL7,PtrAIL8))),((AtBBM/AtPLT4,AtAIL1),(EgAIL2/EgPLT2,(AtPLT2,(AtPLT1,(PtrAIL1A/PtrPLT1A,PtrAIL1B/PtrPLT1B)))))),((((PpAIL1,PpAIL2),(PpAIL3,PpAIL4)),SmAIL1),(AtANT,(EgAIL1B/EgANT1B,(EgAIL1C/EgANT1C,(EgAIL1A/EgANT1A,((PtrAIL2A/PtrANT2A,PtrAIL2B/PtrANT2B),(PtrAIL2C/PtrANT2C,PtrAIL2D/PtrANT2D)))))))),(AtPLT7/AtAIL7,(EgAIL3/EgPLT3,(AtPLT3/AtAIL6,PtrAIL3/PtrPLT3)))),(EgAIL14,((EgAIL15,PpAIL5),(EgAIL16,((PtrAIL9,PtrAIL10),(PtrAIL11,PtrAIL12)))))),(((((EgAIL13,PtrAIL15),EgAIL12),PtrAIL16),EgAIL11),(((PtrAIL13,PtrAIL14),EgAIL10),(SmAIL7,(SmAIL6,(PpAIL6,PpAIL7)))))),(PpAIL9,(PpAIL8,SmAIL8))),PpAIL10),(((((SmAIL11,SmAIL12),PpAIL11),PpAIL12),(CrAIL1,CrAIL2)),(EgAIL8,(((PtrAIL21,PtrAIL22),EgAIL9),(((PtrAIL19,PtrAIL20),SmAIL10),((EgAIL7,EgAIL6),(SmAIL9,(PtrAIL17,PtrAIL18))))))),(CrAIL3,CrAIL4));
